# Spatial variability in size at maturity of golden king crab (*Lithodes aequispinus*) and implications for fisheries management

**DOI:** 10.1098/rsos.171802

**Published:** 2018-03-07

**Authors:** A. P. Olson, C. E. Siddon, G. L. Eckert

**Affiliations:** 1 Alaska Department of Fish and Game, Juneau, AK 99811, USA; 2 College of Fisheries and Ocean Sciences, University of Alaska Fairbanks, Juneau, AK 99801, USA

**Keywords:** size at maturity, fisheries management, spatial variability, golden king crab

## Abstract

Many crab fisheries around the world are managed by size, sex and season, where males are given at least one opportunity to reproduce before being harvested. Golden king crab (*Lithodes aequispinus*) supports a commercial fishery in Southeast Alaska and legal size is based on growth and maturity information from other parts of their range. Size-at-maturity estimates varied for crabs among seven management areas in Southeast Alaska, where male maturity estimates increased in size with increases in latitude, while maturity estimates across their North Pacific range decreased in size with increases in latitude. Depth, temperature and harvest history were not related to variation observed in male maturity estimates. Management implications from this research include reducing legal size in some areas to maximize harvest potential and increasing in others to allow male crabs the opportunity to reproduce before being harvested. A more conservative strategy would incorporate the largest maturity estimate, thus increasing the legal size which would have a negative impact to the commercial fishery, but allow male crabs the opportunity to reproduce before being harvested. This study shows the importance of understanding how life-history characteristics change over space and the challenge incorporating spatial variability for improved fisheries management.

## 1. Introduction

Minimum size limits are an established fisheries management tool that has been used to help ensure long-term sustainability of fishery stocks. A minimum legal size limit allows for individuals to reproduce and contribute to future populations and prevent recruitment overfishing [1,2]. Legal size is used in male-only crab fisheries in the North Pacific and is primarily based on size at maturity while incorporating other life-history parameters such as growth and moulting frequency. Sex and size-selectivity in crab fisheries allows for long-term reproductive viability and sustainability by allowing a proportion of males from a stock to be harvested, while females are left to continually reproduce [1,3].

Size at maturity in males for some crab species can be estimated by using a breakpoint in the ratio between chela height (CH) and carapace length (CL), also known as size at morphometric maturity (SMM), because claw size in males is one of the secondary sexual characteristics that changes at the onset of morphological maturity [4–7]; while in females, size at 50% maturity (SAM) is estimated by examining the presence of embryos or embryo cases attached to the pleopods [8,9]. Examining size at maturity in both males and females may inform how size at maturity may be influenced by fisheries between a harvested and unharvested proportion of a stock within the same species.

Size at maturity can vary within or among geographical areas, leading to challenges in fisheries management. Spatial variability in size at maturity has been observed in high-latitude crab species such as Tanner crab (*Chionoecetes bairdi*) [10], snow crab (*C. opilio*) [11], red king crab (*Paralithodes camtschaticus*) [12], blue king crab (*P. platypus*) [13] and golden king crab (*Lithodes aequispinus*) [9,14]. Size at maturity estimates were generally found to decrease with increasing latitude, except for Tanner crab, which varied by longitude [10,15]. Interestingly, the latitudinal pattern observed of decreased size at maturity is contrary to Bergmann’s rule, which states that animals living in lower latitudes and warmer climates are generally smaller than animals living in higher latitudes and colder climates [16]. Spatial variation in size at maturity for crab species in the North Pacific has been hypothesized to be dependent upon (i) temperature, which is associated with metabolic rate, growth rate, moult timing and moult increment, (ii) size-selective historical fishing pressure [10,15,17,18] and (iii) genetic variation [11].

Temperature and food availability are key environmental variables that can influence size at maturity. In ectotherms, cooler temperatures retard growth and delay maturity, causing individuals to mature at a larger size compared with species in warmer environments [19]. Growth rate and size at maturity are also influenced by food availability, in which increased food availability can result in earlier maturation due to increased amounts of reserved energy [20]. Whether temperature, food availability or their interaction is most influential on size at maturity continues to be debated among scientists and is commonly known as the Berrigan–Charnov puzzle [20,21]. Size selectivity of fishing gear may remove faster growing individuals, while allowing for slow-growing individuals to avoid harvest and reproduce, which can delay the age an individual reaches maturity [22,23]. If growth rate is heritable, this resultant evolutionary effect can decrease population growth, greatly impeding recovery in an overfished population and lead to fishery closures [24]. Evidence of this effect exists for many species, including Pacific salmon (*Salmonidae*) [23] and gastropods in Southern California [25]. However, the effects of fishing pressure on SAM have not been examined in many commercially important species, especially crab.

Golden king crabs are widely distributed across the North Pacific and biological information is limited to help inform fisheries management. Golden king crab life-history attributes include: morphometric maturity at approximately 8 years, a 20 month reproductive cycle, lecithotrophic larvae that remain at depth, mature females that moult approximately every 1.5–2 years and mature males that moult approximately every 10–33 months [26–29]. Slow growth and late maturation make golden king crabs vulnerable to overfishing. Golden king crabs inhabit a very inaccessible environment distributed across the North Pacific at depths ranging from 100 to 1000 m making it difficult to obtain biological information to inform fisheries management [8,9,30].

**Figure 1.**
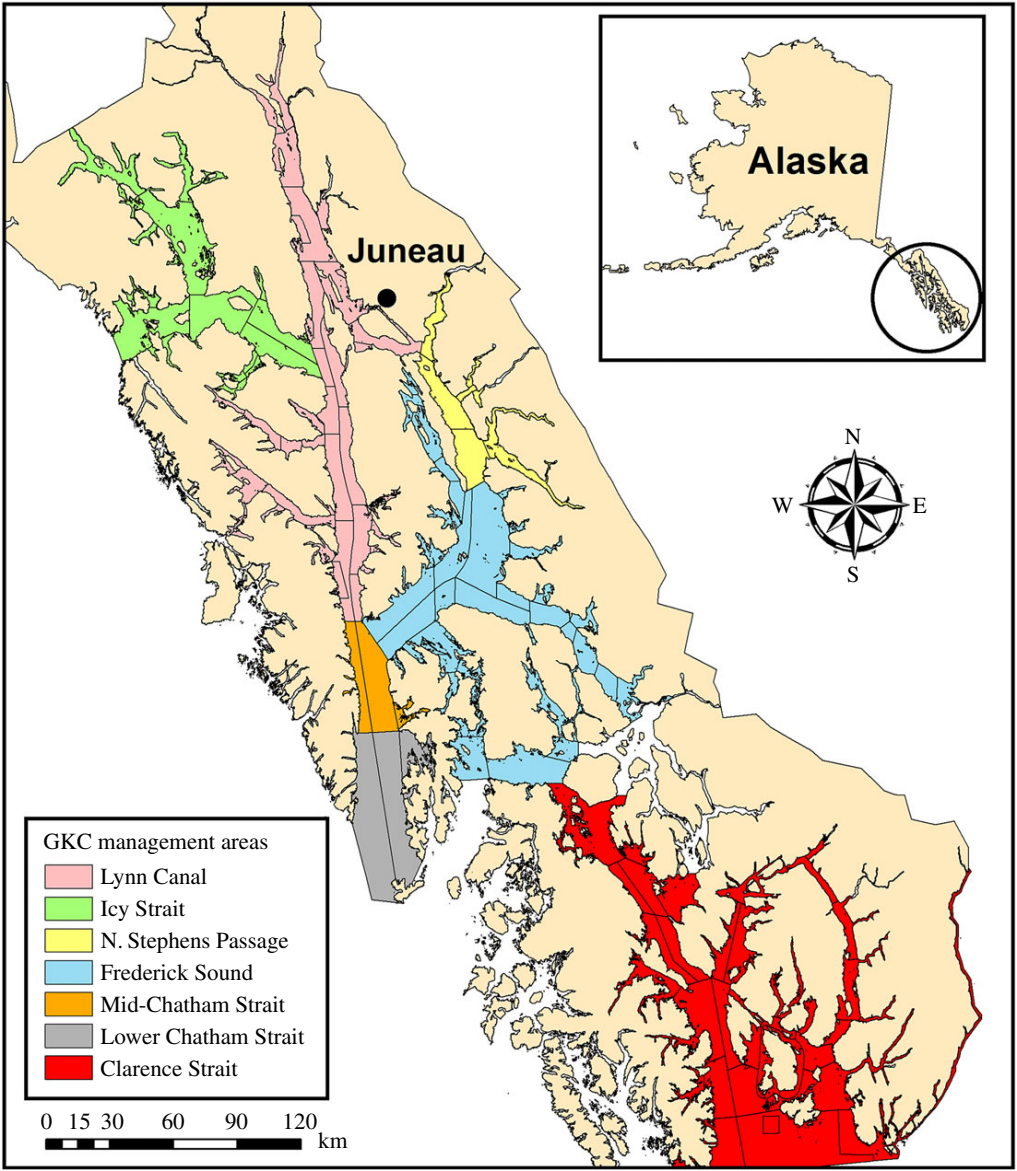
Commercial fishery management areas of golden king crabs in Southeast Alaska. The Northern, East Central and Southern management areas are referred to as Lynn Canal, Frederick Sound and Clarence Strait, respectively, to reflect locations of golden king crab populations geographically.

The golden king crab commercial fishery developed in the early 1980s after the collapse of the red and blue king crab commercial fishery in Alaska (Aleutian Islands, eastern Bering Sea and Southeast Alaska) [8,9,31,32]. In Southeast Alaska, the golden king crab fishery rapidly developed with harvest reaching a historic level in 1987 (461 t) and in 2010 (332 t) and has experienced a period of collapse in the 1990s and is currently in a collapsed state with a harvest of 107 t in 2014 [29]. Management of the Southeast Alaska golden king crab commercial fishery is conducted based on a 3-S (sex, size and season) management system and has further developed by limiting the number of participants, establishing a catch quota per management area and allowing management to close areas if there are stock health concerns. The fishing season typically occurs from February to May or until the catch quota is obtained [29]. The current management areas for the Southeast Alaska golden king crab fishery consist of Lynn Canal, Icy Strait, North Stephens Passage, Frederick Sound, Mid-Chatham Strait, Lower Chatham Strait and Clarence Strait (figure 1). A male-only harvest is allowed using a minimum legal size of 177.8 mm/7.0 in carapace width (CW), intended to allow crabs to mature and reproduce at least once before being harvested.

The historical process on setting the minimum harvest size limit in Southeast Alaska is not very clear; however, information on a variety of sources was considered. The minimum harvest size limit for male golden king crabs was originally based on growth and maturity information for red king crabs from the Gulf of Alaska, which is a different species from a different location [33,34], due to the lack of biological information on golden king crabs. This size limit was later justified using biological data on golden king crab life history [29] as it became available, which consisted of growth information primarily from Frederick Sound and Lynn Canal in Southeast Alaska using a tagging study [31] and SMM estimates most probably from the southeastern Bering Sea (130.0 mm CL) [9]. While SMM and growth are the main components of legal size, spine lengths on a crab’s carapace play a critical role in determining if a specimen is legal for harvest and also requires a closer look. Spine length is included in harvest size limits, and thus variability in spine length could influence the impact of size limits on a fished population. Many invertebrate species, such as the red sea urchin (*Strongylocentrotus franciscanus*) [35], mud crab (*Rhithropanopeus harrisii*) [36] and blue crab (*Callinectes sapidus*) [37], have increased survival attributed to spine morphology that reduces predation risk [38]. Potential predators avoid prey with longer spines due to their unpalatability, and spine length can be plastic and respond to predation pressure [35,37,38]. How spine length varies in golden king crabs remains unknown.

In Southeast Alaska, golden king crab size at maturity has not been estimated and information on basic life-history parameters specific to Southeast Alaska is needed to ensure that fisheries management reflects the regionally specific biology. In this study, we estimate spatial variability in SMM and SAM, the influence of temperature, depth and harvest on spatial variation in SMM estimates, and mean spine contribution to legal-sized males with the overall goal to better inform fisheries management to sustainably manage the golden king crab fishery.

## 2. Material and methods

### 2.1. Size at maturity

Alaska Department of Fish and Game (ADF&G) observers measured golden king crabs during the commercial fishery (February–May) in Southeast Alaska. Male golden king crab CL and CH were measured to the nearest 1 mm during 2007–2014 (*n*=9786). Female CL was measured and pleopods inspected to determine maturity during 1998–2015 (*n*=14 242). Crabs that were visually infected with *Briarosaccus auratum* (*n*=448) were excluded from the analysis, because they are known to suppress growth, potentially hindering maturity [39–41]. Observer coverage included at least one trip in one of the seven management areas in Southeast Alaska each year (figure 1). Male SMM was estimated by fitting a piece-wise linear regression model to the relationship between CL (independent variable) and CH (dependent variable) by least squares. Male SMM was estimated as the breakpoint of the following model:

2.1 $$y=\beta_{0}+\beta_{1}(X)+\beta_{2}{\left(X-C\right)}^{+}+\epsilon ,$$

where *y*=CH (mm); *β*_0_, *β*_1_ and *β*_2_ are estimated regression parameters, *X*=CL (mm); *C* represents the breakpoint; the term (*X*−*C*)^+^ takes the value of zero if the argument is negative and the value of the argument if it is positive; and *ϵ* is the error term. Parameters were estimated using the statistical program R v. 3.1.3 [42] and the extension package SiZer: Significant Zero Crossings [43], and 95% confidence intervals for the breakpoint and the slope parameters were obtained using a bootstrap procedure. One thousand bootstrap samples were taken by resampling the raw data points and refitting the piece-wise linear regression model to calculate SMM for each of the 1000 estimates [44]. We compared SMM among management areas using the Fisher–Behrens statistic:

2.2 $$z=\frac{|\hat{C_{1}}-\hat{C_{2}}|}{\sqrt{s_{1}^{2}+s_{2}^{2}}},$$

where *C*_1_ and *C*_2_ are size at morphometric maturity estimates from different areas, and *s*_1_ and *s*_2_ are standard errors associated with these estimates. The Fisher–Behrens statistic was then compared to the critical value of a normal distribution, *z*_1−*α*/2_, adjusted for multiple tests using a Bonferroni correction, which replaces *α* with *α*/*n*, where *α*=0.05 and *n*=total number of hypotheses to be tested (21) [45,46].

Female SAM was determined based on the presence of embryos or empty embryo cases attached to the pleopods [8,9]. Maturity was estimated by fitting a logistic regression model to the proportion of mature females at a given CL (mm):

2.3 $$P=\frac{1}{1+e^{-a(CL-b)}},$$

where *P* is the predicted proportion of mature females at a given CL (mm), and *a* and *b* are estimated parameters. Parameter *a* represents the shape of the curve and parameter *b* is the SAM CL (mm). The negative log-likelihood was estimated at each maturity proportion as

2.4 $$-\log_{e}\mathrm{CL}=-[M\log_{e}(P+0.0001)+I\log_{e}(1-P+0.0001)],$$

where *M*=number of mature crabs and *I*=number of immature crabs at a given CL (mm). The Excel SOLVER feature was used to find the values of parameters *a* and *b* that minimize the sum of the negative log-likelihoods [47]. The fitted logistic model was then evaluated to determine the CL that corresponds with 50% SAM [4], and 95% confidence intervals were determined using bootstrapping with 1000 replicates, implemented in the statistical program R v. 3.1.3 [4,42]. To determine if SAM differed significantly among areas, a log-likelihood ratio test was used [46,48]:

2.5 $$X^{2}=2[(-\log_{e}{\mathrm{CL}}_{A})-(-\log_{e}{\mathrm{CL}}_{N})],$$

where CL_A_ is the area-specific model with a SAM estimate from a given area and CL_N_ is the non-area-specific model that estimates maturity for the combined data and is compared to the area-specific model.

Male SMM estimates and female SAM estimates from their geographic distribution from the published literature (Japan, eastern Russia, eastern Bering Sea, Aleutian Islands and British Columbia, Canada) as well as from the seven management areas in Southeast Alaska were analysed together and separately (only Southeast Alaska management areas) as a function of latitude using a linear regression model:

2.6 $$y=\beta_{0}+\beta_{1}x+\epsilon ,$$

where *y*=size at maturity estimates (male SMM or female SAM), *β*_0_ and *β*_1_ are estimated regression parameters, *x*=mean latitude (decimal degrees) and *ϵ*=error terms. Size at morphometric maturity for male golden king crab in Prince William Sound, Alaska [49] was estimated using a method that differed from those used in the rest of the literature and, because of this, were excluded from the analysis.

Because removals of males in the fishery could result in variation in SMM estimates, we examined variation in commercial harvest using ADF&G landing data from management areas to determine if fishery harvest was related to variation observed in SMM estimates. Effects of fishery harvest on male SMM estimates from Southeast Alaska were analysed using linear regression models by comparing the total harvest for each management area from two different time periods, 1972–2014 and 2005–2014, to maturity estimates using equation (2.6) where *y*=size at maturity estimates for male crabs from each management area, *β*_0_ and *β*_1_ are estimated regression parameters, *x*=total harvest (*t*) from 1972–2014 or 2005–2014 for each management area, and *ϵ*=error terms.

### 2.2. Environmental variability

Depth and temperature, which may vary across management areas and influence variation in SMM for golden king crabs, were measured from February to May during commercial fishing seasons. ADF&G observers recorded crab pot depth from 1998 to 2014 from fishing vessel sonar measurements, which ranged from 40 to 750 m (*n*=6804) (table 1).

**Table 1.** Sample size (*N*_*D*_) and years that pot depth was measured during the golden king crab commercial fishery in Southeast Alaska.

location	year	*N*_*D*_
Lynn Canal (LyC)	2001–2002, 2004 and 2007–2014	1480
Icy Strait (IS)	2001, 2007–2012 and 2014	615
North Stephens Passage (NSP)	2002, 2004, 2007–2012 and 2014	607
Frederick Sound (FS)	2000–2004, 2007–2008, 2010–2011 and 2013–2014	1445
Mid-Chatham Strait (MC)	2000–2003 and 2007–2014	1329
Lower Chatham Strait (LC)	2000–2001 and 2008–2011	761
Clarence Strait (CS)	1998–2000 and 2014	567

To determine if pot depth varied significantly among management areas and could help explain differences in SMM, mean pot depths were determined for each management area. Mean pot depth was then evaluated by fitting a one-way analysis of variance (ANOVA) and compared among management areas using Tukey’s honest significant difference (HSD) test for pairwise comparisons (*α*<0.05) using JMP 11.0.0 statistical software [50] using equation (2.6) where *y*=SMM, *β*_0_ and *β*_1_ are estimated regression parameters, *x*=mean depth and *ϵ*=error terms.

During 2008–2014, temperature was measured to the nearest 0.2^°^C every hour (*n*=5796) using temperature sensors (HOBO TidbiTs Data Loggers, Onset Computer Co.) attached to crab pots (*n*=40) (table 2). Temperature sensor deployment ranged from days to several weeks, dependent upon the length of an observer trip.

**Table 2.** Sample size (*N*_*T*_) and timing of temperature data recorded hourly at depth (m) during the golden king crab commercial fishing season in Southeast Alaska. Temperature samples at depth were filtered based on set and haul times of pots during the commercial fishery.

location	year	months	*N*_*T*_	mean depth (m)	s.d.
LyC	2008, 2010 and 2012	Feb–Apr	1080	344.4	144.1
IS	2008, 2010 and 2012	Feb	305	282.4	124.5
NSP	2010 and 2014	Mar–May	1346	258.3	70.9
FS	2010 and 2013–2014	Feb–Mar	894	352.6	55.6
MC	2008–2011	Mar and May	1090	488.8	109.4
LC	2010 and 2011	Apr	265	560.2	20.1
CS	2014	Mar–Apr	816	345.6	23.3

To determine whether temperature varied significantly among management areas, mean temperatures were compared using standard deviations. Pot depth and temperature were then used to determine if they were related to SMM for male golden king crabs for all management areas by fitting a linear regression model for depth and temperature using month as a covariate for the temperature analysis using equation (2.6) where *y*=size at morphometric maturity, *β*_0_ and *β*_1_ are estimated regression parameters, *x*=mean depth, and *ϵ*=error terms and,

2.7 $$y=\beta_{0}+\beta_{1}x_{1}+\beta_{2}x_{2}+\epsilon ,$$

where *y*=size at morphometric maturity; *β*_0_, *β*_1_ and *β*_2_ are estimated regression parameters; *x*_1_=mean temperature; *x*_2_=month; and *ϵ*=error terms.

Because temperature data were opportunistically collected by fishery observers and varied by which months and years were sampled across management areas, we examined seasonal and yearly variation in near-bottom temperature at 250 m, a depth representative of golden king crab habitat, using a long-term time series from a mooring station in the northern Gulf of Alaska. The temperature data were retrieved from a publicly available database maintained by the University of Alaska Fairbanks School of Fisheries and Ocean Sciences (http://www.ims.uaf.edu/gak1/). The Gulf of Alaska (GAK1) mooring station is located adjacent to the coast near Seward (59^°^50.7′ N, 149^°^28.0′ W) at a depth of 250 m. Temperature measurements at 250 m, recorded every quarter hour (*n*=200 299), were averaged by month during 2008–2013 (January–December, *n*=72). Mean monthly temperatures from GAK1 (*n*=72) were analysed for assumptions of normality (Shapiro–Wilk test *p*>0.05) and homogeneity of variances (Bartlett test *p*>0.05) and were evaluated by fitting a one-way ANOVA and compared using Tukey’s HSD pairwise comparisons (*α*<0.05). Then monthly mean temperatures for GAK1 and Southeast Alaska (GAK1: February–May *n*=6 for each month and Southeast Alaska: February *n*=7, March *n*=9, April *n*=5 and May *n*=2) were analysed for assumptions of normality (Shapiro–Wilk test *p*>0.05) and homogeneity of variances (Levene’s test *p*>0.05) and were evaluated by fitting a two-way ANOVA and compared using Tukey’s HSD pairwise comparisons (*α*<0.05). These analyses were used to determine (i) consistency in temperature at depth across months and (ii) whether opportunistic intermittent sampling by fisheries observers (February–May from 2008 to 2014) are representative of those measured consistently in the northern Gulf of Alaska.

### 2.3. Crab size and spine contribution

We investigated the CW, CL and spine contribution to size of legal male golden king crab to determine variation among management areas and to develop growth equations for each management area of Southeast Alaska. ADF&G observers and port samplers sampled legal-sized males during 2013–2014 (*n*=1592) and measured CL and CW to the nearest 1 mm with and without spines at the widest point of the carapace. Mean spine contribution was calculated as the difference in CW with and without spines and includes contribution of spines on both sides of the carapace.

### 2.4. Legal size

To reassess the current legal size in each of the management areas in Southeast Alaska, estimates of mean spine contribution, SMM and an area-wide growth increment (16.3 mm CL, s.d.=3.0, *n*=45 from Lynn Canal, Icy Strait and Frederick Sound) [31] were used to generate a spatially specific legal size. These metrics were then used in conjunction with CL and CW measurements to develop a CL–CW model in all seven management areas to determine if golden king crab males have at least one opportunity to reproduce at least once before being harvested under the fishery legal size of 177.8 mm/7.0 in CW. The relationship between CL and CW was used in order to convert CL measurements (morphometric maturity and moult increment) into CW because the commercial fishery uses CW for legal size determinations:

2.8 CW=mCL−b,

where CW is given carapace width (mm), CL is a given carapace length (mm), *m* is the slope and *b* is the CW intercept. Equation (2.8) was then modified to incorporate SMM estimates, a moult increment and mean spine contribution:

2.9 $$\mathrm{CW}=(m({\mathrm{SMM}}_{a}+\mathrm{moult}\;\mathrm{increment})-b)+{\mathrm{mean}\;\mathrm{spine}\;\mathrm{contribution}}_{a},$$

where SMM is the size at morphometric maturity estimate, *a* is the area of interest and *b* is the CW intercept from equation (2.9), and mean spine contribution.

Additionally, port samplers measured the CL of harvested legal male golden king crabs (*n*=93 075) during the commercial fishery from 1972 to 2014 and the data were used to determine historical harvest loss by adjusting the current legal size (177.8 mm/7.0 in CW) to reflect area-specific legal size estimates and applying the largest area-specific legal size estimate for all management areas using equation (2.8), respectively, for each area to convert CL to CW.

## 3. Results

### 3.1. Size at maturity

**Figure 2.**
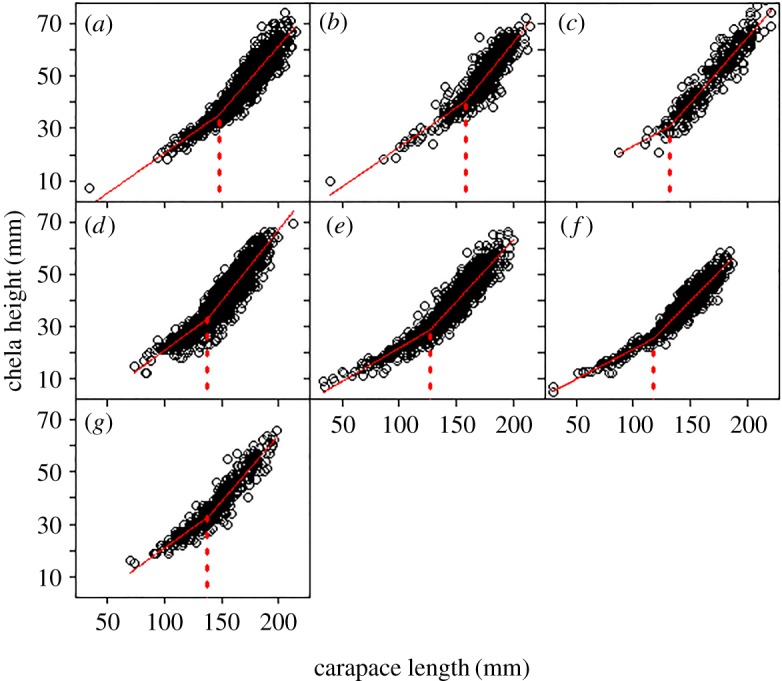
Size at morphometric maturity (CL) for male golden king crabs in Southeast Alaska by management area. Estimates include: (*a*) Lynn Canal (147.3 mm), (*b*) Icy Strait (158.0 mm), (*c*) North Stephens Passage (131.9 mm), (*d*) Frederick Sound (137.6 mm), (*e*) Mid-Chatham Strait (127.3 mm), (*f*) Lower Chatham Strait (117.9 mm) and (*g*) Clarence Strait (138.5 mm).

**Figure 3.**
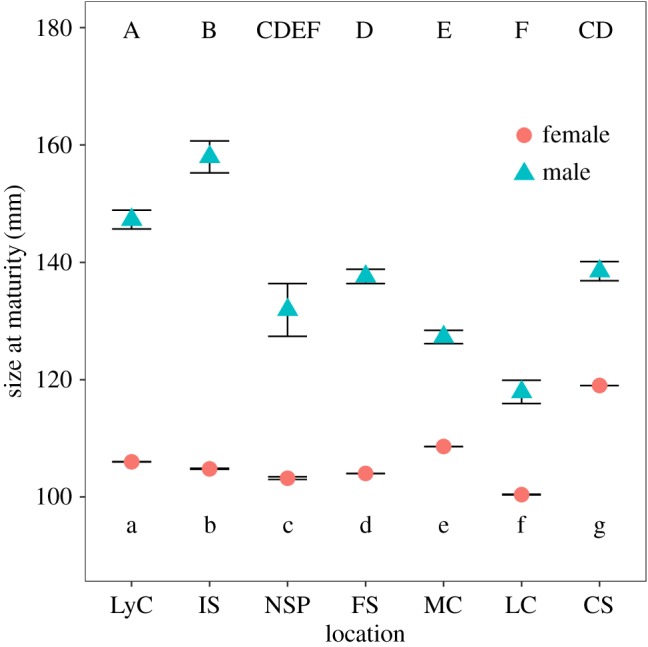
Comparisons of maturity estimates ±s.e. for male (triangles) and female (circles) golden king crab in Southeast Alaska. Significant differences among areas are represented by capital letters (males) at the top and lower case letters (females) at the bottom.

**Figure 4.**
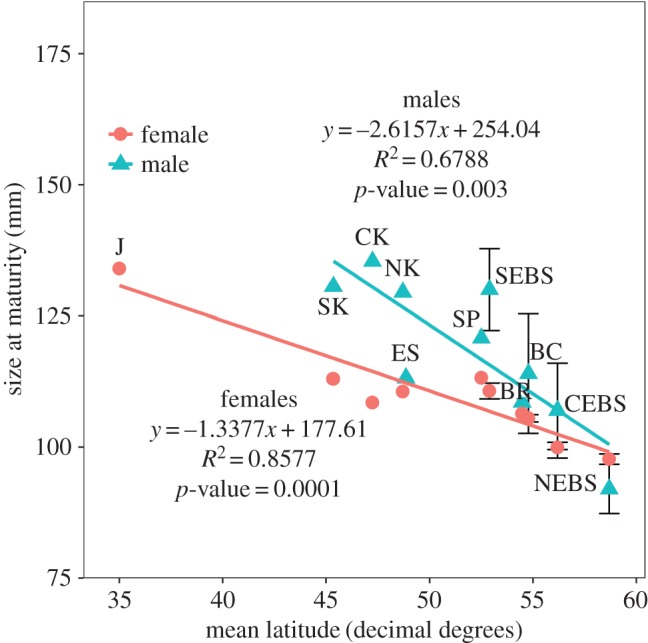
Latitudinal variation in SMM and SAM for golden king crabs from the published literature. Male maturity estimates were determined using SMM and female maturity estimates were determined using SAM. Maturity estimates from the published literature include: Japan (J) [8,51]; the southern, central and northern Kuril Islands (SK, CK and NK) [14], and eastern Sakhalin Island (ES) [52], Russia; Seguam Pass (SP), Bowers Ridge (BR) [32]; south, central and northeastern Bering Sea (SEBS, CEBS and NEBS) with 95% confidence intervals for males [10] Alaska; and Canada (BC) with 95% confidence intervals [8]. Note that the Japanese estimated SAM for females reflects the estimated size at which 60% of the females were found to be mature [8,51]. A linear regression line for male (blue) and female (red) golden king crabs is plotted to represent the strength in the latitudinal cline across Japan, eastern Russia, the Bering Sea, Aleutian Islands and Canada. Both male (*R*^2^=0.677) and female (*R*^2^=0.858) golden king crabs exhibited strong patterns in latitudinal variation with SMM and SAM estimates decreasing as latitude increased.

Male SMM estimates varied significantly among management areas, with the largest difference of 40.1 mm occurring between Icy Strait and Lower Chatham Strait. Icy Strait crabs had the largest SMM estimate (158.0 mm CL), followed by Lynn Canal (147.3 mm CL), Clarence Strait (138.5 mm CL), Frederick Sound (137.6 mm CL), North Stephens Passage (131.9 mm CL), Mid-Chatham Strait (127.3 mm CL) and Lower Chatham Strait (117.9 mm CL) (figure 2 and table 3). Significant differences were found among many of the management areas (figure 3), with the exception of North Stephens Passage compared to Frederick Sound, Mid-Chatham Strait, Lower Chatham Strait and Clarence Strait, and Frederick Sound compared to Clarence Strait. Female golden king crab SAM estimates were all significantly different among management areas (*p*<0.001), but did not follow the same spatial variability pattern as observed in males (figure 4). Clarence Strait female crabs had the largest maturity estimate (119.0 mm) followed by Mid-Chatham Strait (108.6 mm), Lynn Canal (106.2 mm), Icy Strait (104.8 mm), Frederick Sound (104.0 mm), North Stephens Passage (103.2 mm) and Lower Chatham Strait (100.4 mm) (figure 3 and table 3).

**Table 3.** Maturity estimates for male (*N*) and for female (*N*) golden king crab collected during the commercial fishery in Southeast Alaska with 95% confidence intervals and mean latitude (decimal degrees) where crab were captured.

	mean latitude				males				females	
location	(decimal degrees)	s.e.	years	N	SMM (mm)	95% CI	years	*N*	SAM (mm)	95% CI
LyC	58.230	0.009	2007, 2009–2013	1859	147.3	134.0–150.7	2001, 2004, 2007–2015	1742	106.2	103.7–108.3
IS	58.154	0.004	2007–2012, 2014	668	158.0	149.9–162.7	2001, 2003, 2007–2009, 2011–2012, 2014	929	104.8	102.2–106.3
NSP	57.901	0.008	2007–2012, 2014	398	131.9	120.3–181.0	2002, 2004, 2007–2012, 2014	1192	103.2	102.2–104.2
FS	57.179	0.003	2007–2008, 2010–2011, 2013–2014	2294	137.6	131.0–141.0	2000–2004, 2007–2008, 2010–2011, 2013–2014	4370	104.0	103.4–104.6
MC	56.827	0.003	2007–2013	2183	127.3	121.4–131.4	2000–2003, 2007–2014	3681	108.6	107.8–109.4
LC	56.308	0.005	2008–2011	1630	117.9	115.4–121.6	2000–2001, 2008–2011	1048	100.4	98.4–102.2
CS	55.830	0.009	2011–2013	754	138.5	130.0–143.0	1998–2000, 2011–2014	1280	119.0	117.6–120.5

**Figure 5.**
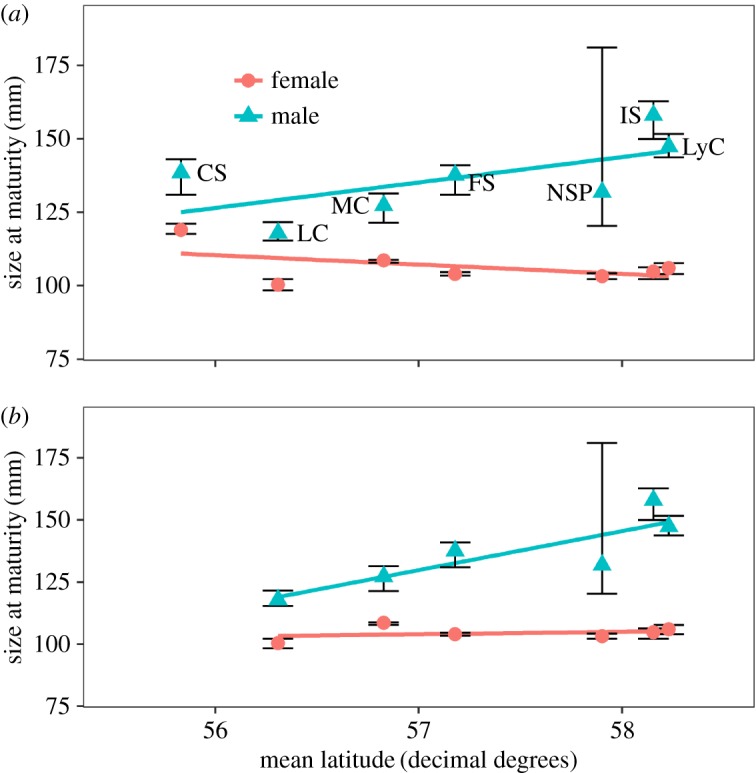
Latitudinal variation in SMM and SAM golden king crabs in Southeast Alaska. Estimates have 95% confidence intervals (bars) for male (triangles) and female (circles) golden king crab in Southeast Alaska. Estimates include: LyC, IS, NSP, FS, MC, LC and CS with a linear regression (*a*) including all areas and (*b*) with CS removed. Location abbreviations defined in table 1.

Maturity estimates of golden king crabs decreased with increasing latitude over their geographical distribution when excluding Southeast Alaska, for both males (*F*=16.75, *p*-value=0.003, *R*^2^=0.677) and females (*F*=48.22, *p*-value=0.0001, *R*^2^=0.858) (figure 4). Male SMM in Southeast Alaska was not related to latitude when the most southerly area, Clarence Strait, was included in the analysis (*F*=3.092, *p*-value=0.139, *R*^2^=0.382) (figure 5*a*). However, when Clarence Strait was removed from the analysis, SMM increased with increasing latitude (*F*=10.94, *p*-value=0.0297, *R*^2^=0.665) (figure 5*b*). For females latitude was not related to SAM (*F*=1.64, *p*-value=0.256, *R*^2^=0.247) (figure 5*a*), even when crabs from Clarence Strait were removed from the analysis (*F*=0.310, *p*-value=0.586, *R*^2^=0.080) (figure 5*b*). Interestingly, for Clarence Strait both male SMM and female SAM estimates did not fit the increasing latitudinal cline observed in Southeast Alaska.

Fishery harvest varied among management areas, but was not related to variation in SMM. Total harvest during 1972–2014 was highest in Frederick Sound (3671 t) and Lynn Canal (2054 t), followed by Mid-Chatham Strait (1499 t), Icy Strait (741 t), North Stephens Passage (306 t), Lower Chatham Strait (304 t) and Clarence Strait (161 t). Total harvest from 1972 to 2014 did not explain the variation observed in male golden king crab SMM estimates for Southeast Alaska (*F*=0.1624, *p*-value=0.704, *R*^2^=0.032). Additionally, harvest from a more recent time period (2005–2014) did not explain the variation observed in SMM estimates (*F*=0.205, *p*-value=0.670, *R*^2^=0.039). Total harvest from 2005–2014 was highest in Frederick Sound (1131 t) and Lynn Canal (639 t) followed by Mid-Chatham Strait (427 t), Icy Strait (211 t), North Stephens Passage (79 t), Clarence Strait (76 t) and Lower Chatham Strait (65 t).

### 3.2. Environmental variability

The mean depth of pots sampled during the commercial fishery varied significantly among management areas with five groupings (table 4). Pots sampled in Mid-Chatham Strait and Lower Chatham Strait were the deepest, with mean depths below 500 m, followed by Lynn Canal and Clarence Strait with depths approximately 350 m; while mean depth of pots in Frederick Sound, Icy Strait and North Stephens Passage ranged from 240 to 305 m (table 4).

**Table 4.** Mean depth of golden king crab habitat in Southeast Alaska where crabs were harvested.

location	mean depth (m)	s.d.	Tukey’s HSD
LyC	346.0	101.3	B
IS	277.2	70.3	D
NSP	246.2	41.1	E
FS	305.5	86.1	C
MC	504.1	118.9	A
LC	514.0	72.1	A
CS	357.0	83.7	B

**Figure 6.**
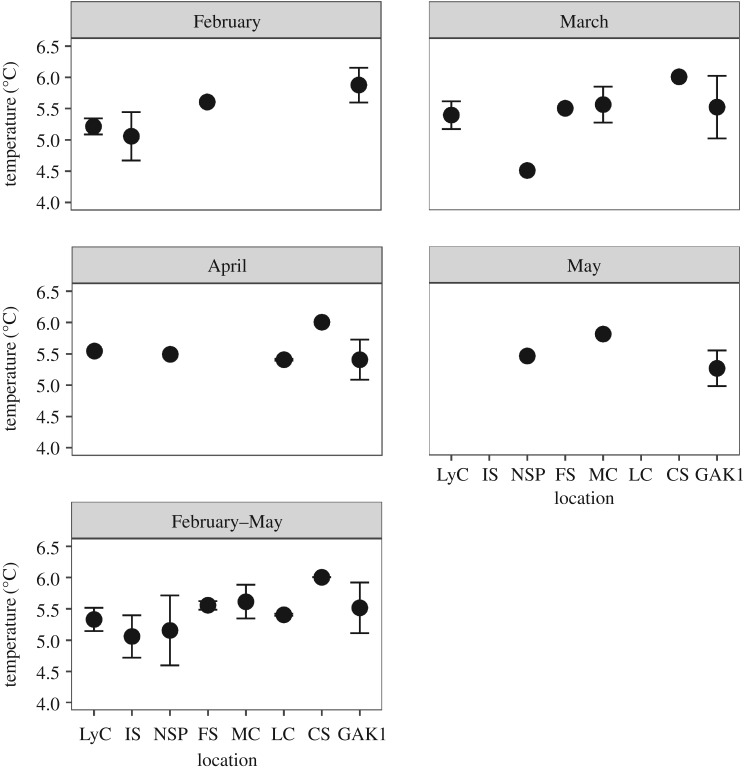
Mean ± s.d. monthly temperatures at depth among Southeast Alaska golden king crab management areas and the Gulf of Alaska mooring station (GAK1) (Feb–May).

**Figure 7.**
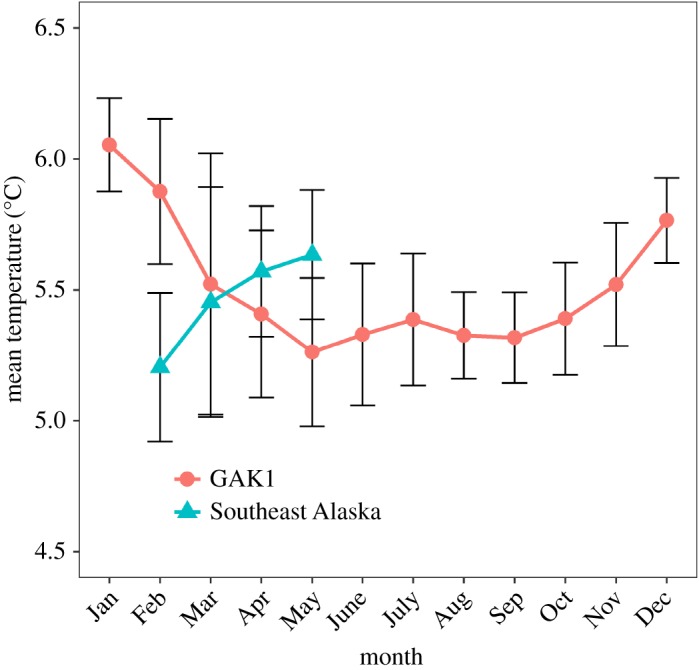
Mean ± s.d. monthly temperature from GAK1 and Southeast Alaska golden king crab management areas by month. Temperature sampled at 250 m depth at GAK1 and from 246 to 514 m depth in Southeast Alaska.

The mean monthly bottom temperature sampled during the golden king crab commercial fishery (February–May) varied within a range of 5.1–6.0^°^C among management areas and the GAK1 mooring station (figure 6). Monthly mean temperatures for GAK1 throughout the year ranged from a low of 5.3^°^C in May, June, August and September to a high of 6.1^°^C in January (figure 7). When GAK1 and pooled Southeast Alaska temperatures were analysed for monthly variation between locations during the timing of the golden king crab commercial fishery, there was a significant interaction between location and month (*F*=4.19, *p*-value=0.01). During the month of February, temperatures measured in Southeast Alaska were significantly cooler than at GAK1 (*p*-value=0.0013), but these differences did not occur in March, April or May (figure 7). Additionally, SMM was not related to variation in depth across management areas (*F*=1.778, *p*-value=0.240, *R*^2^=0.262) and did not relate to variation in mean monthly temperature by management area from Southeast Alaska (*F*=0.492, *p*-value=0.5, *R*^2^=0.0394) and month was a non-significant covariate (*p*-value=0.979).

### 3.3. Crab size and spine contribution

Mean spine contribution to legal size measurements varied among management areas, but the magnitude of the differences was very small and not likely biologically relevant (approx. 1 mm). Clarence Strait had the largest spine contribution (10.1 mm) followed by North Stephens Passage (10.0 mm), Lower Chatham Strait (9.6 mm), Lynn Canal (9.2 mm), Mid-Chatham Strait (9.0 mm), Frederick Sound (8.9 mm) and Icy Strait (8.9 mm). Clarence Strait mean spine contribution to legal size was larger than Lynn Canal, Mid-Chatham Strait, Frederick Sound and Icy Strait (table 5). North Stephens Passage and Lower Chatham Strait mean spine contribution to legal size were larger than Mid-Chatham Strait, Frederick Sound and Icy Strait (table 5).

**Table 5.** Mean spine contribution (mm) to legal-sized male golden king crabs in Southeast Alaska.

		mean		mean spine contribution	
location	*N*	CW (mm)	s.d.	to legal size (mm)	s.d.
LyC	187	200.9	12.3	9.2	2.8
IS	121	200.0	13.6	8.9	2.1
NSP	152	196.2	17.0	10.0	2.9
FS	623	181.6	11.8	8.9	2.6
MC	268	184.1	11.0	9.0	3.2
LC	70	176.6	7.8	9.6	3.2
CS	171	183.4	11.7	10.1	2.8

### 3.4. Legal size

Using the CL–CW relationship for each area and incorporating SMM estimates, growth increment from Southeast Alaska (16.3 mm CL) and mean spine contribution to legal size, and assuming that reproduction occurs after moulting, we calculated that crabs are capable of reproducing at least once before being harvested and reaching the current legal size (177.8 mm CW) in five out of seven areas (table 6). Projected area-specific legal size estimates (CW) for Clarence Strait (177.6 mm), Frederick Sound (173.4 mm), North Stephens Passage (168.8 mm), Mid-Chatham Strait (163.2 mm) and Lower Chatham Strait (159.8 mm) remain under the legal size. Projected area-specific legal size estimates (CW) for Icy Strait (196.5 mm) and Lynn Canal (186.0 mm) substantially exceeded the legal size (177.8 mm). Area-specific CL–CW models were significant for all areas (*p*-value<0.001), where North Stephens Passage had the strongest relationship between CL and CW (*n*=152, *F*=1803, *R*^2^=0.92), followed by Frederick Sound (*n*=623, *F*=4362, *R*^2^=0.88), Icy Strait (*n*=121, *F*=818.5, *R*^2^=0.87), Clarence Strait (*n*=171, *F*=995.6, *R*^2^=0.85), Lynn Canal (*n*=187, *F*=1064, *R*^2^=0.85), Mid-Chatham Strait (*n*=268, *F*=1306, *R*^2^=0.83) and Lower Chatham Strait (*n*=70, *F*=136.4, *R*^2^=0.67).

**Table 6.** Projected area-specific legal size (carapace width (CW)) of male golden king crab in Southeast Alaska. Incorporating SMM, growth increment per moult (CL 16.3 mm) [31] and mean spine contribution for each area (tables 3 and 5) (equations (2.8) and (2.9)) resulted in a projected area-specific legal size CW (mm) for comparison to the current legal size (177.8 mm CW).

	size at morphometric	area-specific legal	area-specific legal
location	maturity (SMM) CL (mm)	size CW (mm)	size CW (in)
LyC	147.3	186.0	7.3
IS	158.0	196.5	7.7
NSP	131.9	168.8	6.6
FS	137.6	173.4	6.8
MC	127.3	163.2	6.4
LC	117.9	159.8	6.3
CS	138.5	177.6	7.0

Using historical harvest from 1972 to 2014 and adjusting the current legal size (177.8 mm/7.0 in CW) of male golden king crabs to be reflective of area-specific legal size revealed that only Lynn Canal (186.0 mm/7.3 in CW) and Icy Strait (196.5 mm/7.7 in CW) were impacted due to their larger area-specific legal sizes with a historical harvest loss of 288 t and 348 t, respectively (table 7). Adjusting the legal size for all management areas to the largest area-specific legal size (Icy Strait 196.5 mm/7.7 in CW) had the greatest impact on harvest loss in Lower Chatham Strait (84%) and least in Lynn Canal (38%) (table 7).

**Table 7.** Estimated harvest of sub-legal crabs and potential harvest loss in the golden king crab commercial fishery with a change in legal size using area-specific legal sizes and a maximum legal size. Area-specific legal size (CW) incorporates SMM, growth increment and mean spine contribution to legal size. Maximum legal size (7.7 in CW) is the largest size needed, so no single area is overexploited. These legal sizes provide a range of potential historical harvest loss from 1972 to 2014.

				% historical harvest of sub-legal			total harvest loss (t)		
	no. crab	total	area-specific	current	area-specific	max	current	area-specific	max
location	sampled	harvest (t)	legal size (in)	(7.0 in)	legal size	(7.7 in)	(7.0 in)	legal size	(7.7 in)
LyC	16 701	2054	7.3	3	14	38	62	288	781
IS	4473	741	7.7	5	47	47	37	348	348
NSP	7580	306	6.6	5	0	51	15	0	156
FS	46 883	3674	6.8	9	2	74	331	73	2719
MC	12 653	1499	6.4	7	0	66	105	0	989
LC	3354	304	6.3	7	0	84	21	0	255
CS	1431	161	7.0	2	0	56	3	0	801

## 4. Discussion

Spatial variation in size of maturity estimates for male and female golden king crabs varied across management areas in Southeast Alaska, with resultant implications for fishery management to allow crabs to reproduce at least once and thus prevent overharvest. The explanation for spatial variation in SMM estimates of male golden king crab in Southeast Alaska is not immediately apparent, as SMM was unrelated to depth, temperature, harvest pressure and spine length. Estimates of male SMM generally increased with increases in latitude within Southeast Alaska, but the relationship was not significant, while estimates of female SAM did not vary with latitude. This intriguing pattern, which is the opposite of what has been observed over a broad geographical area across the North Pacific and Bergmann’s rule, may result from the glacial fjord system and unique habitats of golden king crabs in Southeast Alaska. Female SAM in Southeast Alaska was smaller than male SMM, and the differences among management areas varied less for females than for males. The small differences across management areas for females suggest that female golden king crab become sexually mature over a narrow size range [9,32]. Interestingly, Aleutian Islands female golden king crab populations sampled in 1981–1984 have a smaller SAM range (106.4 mm–110.7 mm) than those in Southeast Alaska (100.4 mm–119.0 mm). Of the seven management areas within Southeast Alaska, Clarence Strait maturity estimates for male and female crabs did not fit the increasing latitudinal cline observed, indicating that Clarence Strait is a uniquely different area among the other management areas.

Estimates of male golden king crab location-specific mature CWs which incorporated SMM, growth increment and mean spine contribution to legal size in Southeast Alaska revealed that the current legal size of 177.8 mm/7.0 in CW may not allow crabs to reproduce at least once before being harvested in two of the seven management areas: Icy Strait and Lynn Canal. This indicates that the current legal size for the golden king crab commercial fishery in Southeast Alaska could be improved to incorporate area-specific maturity; however, a revised management approach is not clear. Adoption of area-specific legal sizes for each management area would be ideal as it would maximize harvest for each area, but this is not practical because fishermen harvest from multiple management areas at the same time, thus it would be difficult to enforce. The most conservative option would be to increase the legal size throughout the region to exceed the largest area-specific legal size CW (Icy Strait (196.5 mm/7.7 in CW)), so that no single area is potentially overexploited. If the legal size was increased to 196.5 mm/7.7 in CW, the potential harvest loss based on historical harvest from 1972 to 2014 would differ across management areas, with greatest harvest loss in Lower Chatham (84%) and least in Lynn Canal (38%). To alleviate the high potential loss of harvest in Lower Chatham Strait, which has the smallest SMM estimate (117.9 mm CL) and area-specific legal size (159.8 mm/6.3 in CW), an alternative management strategy could be applied by opening this area to a reduced legal size when all other management areas are closed. We did not calculate long-term harvest loss owing to a reduction in reproductive output associated with harvest of males before they had a chance to reproduce. This issue and the recent decline in harvest and large size at morphometric maturities, especially in Icy Strait and Lynn Canal, are worthy of future study.

Male SMM estimates in Southeast Alaska varied spatially among contiguous waterways, including Frederick Sound, Mid-Chatham Strait and Lower Chatham Strait. This variation may indicate that crabs may segregate by size or could be spatially separate populations. By contrast, areas that were spatially separated had crabs with similar SMM; crabs from Clarence Strait were surprisingly not significantly different in SMM from Frederick Sound and North Stephens Passage. Low power resulting from a small sample size and lack of immature crabs may explain this result for North Stephens Passage and more samples may reveal a difference. We initially planned to examine SMM estimates over time for each management area to reveal temporal variability and elucidate mechanisms for variability in size at morphometric maturity. However, variable sampling effort across management areas and years by fishery observers and declines in population levels over the past few years resulted in too small sample sizes when data were subdivided by years.

We could not detect an influence of depth or temperature on SMM for male golden king crabs. Mean depth varied among management areas and ranged from 246 m to 514 m. Mean temperature measured on crab pots and at a long-term mooring at 250 m depth was relatively constant and ranged only over 1^°^C, from 5.1^°^C to 6.1^°^C. Significant differences in mean temperature occurred in February between Southeast Alaska and the mooring at GAK1, suggesting that winter temperatures may be colder in the protected inner waters of Southeast Alaska than offshore in the Gulf of Alaska. Bottom temperature data were only available at the time of the fishery in Southeast Alaska and seasonal variation in temperature at depth could be further investigated. It was surprising that temperature was not related to the variability in male SMM estimates observed for golden king crabs in Southeast Alaska, but this result may arise from the lack of variation in temperature at deep depths.

We found no relationship between total harvest of golden king crabs over two different time periods, from 1972 to 2014 and from 2005 to 2014, and SMM estimates among management areas. We were not able to resolve shorter-term patterns, due to small sample sizes of crabs in any given year. The relationship between maturity and harvest pressure could be further investigated to determine if temporal variability in harvest pressure may have an effect on size at maturity for golden king crab and to better inform current size-at-maturity estimates. The influence of harvest pressure on SMM can have important implications for fisheries management [7,23]. Removing larger and more reproductively viable individuals can result in decreased size at maturity, which then requires an adjustment to harvest strategy to maintain harvest rates and spawning biomass [7]. Reductions in size at maturity can exacerbate population declines, leading to a collapse if fisheries management does not adapt by adjusting minimum legal size and gear selectivity in order to maintain fishery sustainability [24]. The sustainability of the Southeast Alaska golden king crab fishery is questionable, as it has collapsed multiple times; thus monitoring SMM to detect possible temporal changes could potentially inform of future sustainability concerns. If a larger legal size were to be adopted, future studies could investigate if spatial variation in SMM changes over time and across management areas.

A similar study was conducted in Southeast Alaska looking at influences of latitude, temperature, depth and harvest pressure on size-at-maturity estimates for male and female Tanner crabs [53]. Variability in maturity estimates was observed across Southeast Alaska, but did not follow a latitudinal cline as we observed for populations of golden king crab. Interestingly, neither temperature, depth, nor harvest pressure explained the variability observed in Tanner crab maturity estimates, similar to our results here. Siddon & Bednarski [53] attributed the lack of a relationship between these factors and maturity estimates to temperature influences on growth and the limiting effects of food availability. As temperature increases, moulting frequency and overall growth increase in the lab for Tanner crabs and blue crabs [54,55]. When food availability is limited, less energy is available for growth and reproduction, which can cause delays in size at maturation [56,57]. Temperature in golden king crab habitat at depth varied by location, but by less than 1.0^°^C, indicating temperature effects on growth and food availability may be minimal. However, competition for prey resources and predator influences on golden king crab could cause variation in food availability and potential spatial variation in size at maturity, but this hypothesis needs to be further investigated.

Predation may be influential in determining size at maturity through direct and indirect effects. High levels of predation can increase the rate of growth and speed at which a prey species reaches maturity in order to contribute to future populations with the trade-off of having a higher mortality rate [58,59]. On the other hand predators can indirectly influence prey foraging behaviour and through reduced foraging opportunity limit food availability, with the result of a reduced growth rate and ultimately delaying the onset of maturity [60]. A variety of predators consume golden king crabs, such as Pacific halibut (*Hippoglossus stenolepis*), Pacific cod (*Gadus macrocephalus*) and sablefish (*Anoplopoma fimbria*), based on anecdotal reports from the fishing community and observations of stomach contents in processed fish (anonymous fisherman, Petersburg, AK 2015, personal communication). Published information on groundfish predation rates on king crabs is limited; however, an analysis of stomach contents conducted by Livingston [61] from 1981 to 1984 looked at trends in Pacific cod predation on three commercially important crab species in the eastern Bering Sea: red king crabs, snow crabs and Tanner crabs. Female red king crabs only made up a small portion of the Pacific cod diet (1.4–3.8%), while snow crabs and Tanner crabs at age 1 made up a large proportion of their diet (84–95% and 27–57%, respectively) [61]. Similar studies conducted in Kodiak Alaska from 1973 to 1975 looked at groundfish stomachs of Pacific cod (*n*=4000), sculpins (*Myoxocephalus* spp., *n*=320) and yellow Irish lord (*Hemilepidotus jordani*, *n*=535) and found that less than 2% of the stomach contents contained red king crabs, while Tanner crabs were the dominant prey crab species [62]. These results indicate that these groundfish species are not major predators on red king crabs, which is similar in size and morphology to golden king crabs.

Mean spine contribution (mm) to legal-sized male golden king crabs ranged from 8.9 to 10.1 mm and varied across management areas, but whether this variation is biologically meaningful is not clear. The 1.2 mm difference between the largest and smallest estimates does not have any resulting management implications. Studies of hatchery-raised and wild juvenile red king crabs in Southeast Alaska [63] and blue crabs in Maryland [64] suggest that crab spine length can be plastic and becomes larger in the presence of predators. Future studies could investigate the influence of predators on spine lengths for golden king crabs in Southeast Alaska. It is interesting that even though spine length for golden king crabs varied slightly among management areas, the pattern was different from the spatial pattern in SMM variation.

Variable growth rates could influence golden king crab size at maturity. In Southeast Alaska historical data on the mean growth increment per moult lacked sufficient spatial resolution to determine if variability exists across management areas, as data are available from limited areas, primarily from Frederick Sound and Lynn Canal [31]. Growth increment per moult for male golden king crabs in the eastern Aleutian Islands (Amukta, Chagulak and Yunaska) was 14.5 mm CL (*n*=517, s.d.=2.95) [65,66]. Unfortunately, data on growth in the Aleutian Islands were not spatially identified, so spatial resolution to determine variability in growth rates was not available [66]. Growth of golden king crabs greater than 90 mm CL from the Aleutians was relatively constant, indicating that growth rates may stabilize once a certain size is reached [66]. In the Beagle Channel, Argentina, southern king crab (*Lithodes santolla*) growth increment per moult was 11.4 mm CL (*n*=66, s.d.=1.7) [65]. However, other growth studies from the Strait of Magellan and Beagle Channel found that crabs greater than 70 *mm* CL on average had a growth increment of 11.1 mm and 9.3 mm CL, respectively, indicating spatial variability in growth [67,68]. To determine whether growth is spatially variable for golden king crabs across Southeast Alaska, mark–recapture studies could be conducted in different management areas.

Another possible explanation that may be influencing golden king crab growth is the parasite *B. auratum*, previously identified as *B. callosus*, a parasitic barnacle that suppresses growth, feminizes and castrates male crabs and sterilizes female crabs [41]. This parasite has a root system (interna) that runs through the inside of its host taking over their body and produces multiple egg sacs (externa) under the abdominal flap [39]. Because *B. auratum* suppresses growth, it could potentially hinder the size at which crabs become mature, resulting in very few infected crabs that would recruit to the fishery and be removed to prevent further infections [39,69,70]. In Southeast Alaska, from 1998 to 2014 observers documented the highest incidence rate of infection in Clarence Strait (3.7%, *n*=6011), while the remaining management areas combined from 2000 to 2014 had a much lower infection rate (0.3%, *n*=65 043) (A. Olson, personal observation). In the most recent years, from 2011 to 2014, fishery observers documented 176 infected crabs in Clarence Strait, while in contrast, only 27 crabs from the remaining management areas combined were infected.

Research on golden king crab genetics could reveal stock structure and provide management information to create biologically representative management areas to better manage the fishery. Genetic studies of red king crabs in the North Pacific revealed three distinct genetic groupings: (i) Adak Island, (ii) Bering Sea–Gulf of Alaska and (iii) Southeast Alaska, with Southeast Alaska having the lowest levels of genetic diversity and mtDNA, but significant genetic heterogeneity among populations over a small geographic scale [71,72]. This result suggests that the glacial fjord system and enclosed bays of Southeast Alaska may result in decreased connectivity among king crab populations, with potentially limited larval dispersal and limited gene flow resulting in self-recruiting populations [71–73]. The current management areas in Southeast Alaska are based on historical harvest patterns and are derived from statistical areas that are entirely used for salmon management. Thus, the spatial arrangement of these statistical areas dictates possible boundaries for management areas for golden king crabs and may not be representative of biologically distinct stocks. Future genetic studies of golden king crabs could determine whether heterogeneity among management areas exists and inform whether the current management areas in Southeast Alaska truly represent separate stocks.

Sampling bias in size at maturity in this study is possible because crab were caught using pots, which are known to capture a disproportionately greater number of larger mature crab than small immature crab [74], which could result in size-at-maturity estimates that are biased towards mature individuals. One of the management areas in this study, North Stephens Passage, contained few small crabs. This maturity estimate may be biased because the likelihood of obtaining smaller crab in the resampling of the bootstrap procedure was low and resulted in a large upper bound confidence interval. Because all crabs in this study were captured with pots, this sampling bias applies throughout and therefore does not simply explain the spatial variability in maturity estimates observed. Additionally, sampling bias could have been reduced by bootstrapping on the residuals rather than resampling observations. An alternative sampling method, such as trawling, could potentially sample both small and large crabs; but trawling is not feasible due to the narrow channels and rocky shores of the fjord system in Southeast Alaska.

Information on life history is a critical to the successful long-term sustainability and management of a fishery. Parameters such as maturity, growth and reproduction provide a basis for defining fishing seasons, legal size and harvest levels without adversely affecting the reproductive potential of a fished stock. In Southeast Alaska, golden king crab is a prime example of the effect of gaps in our understanding of life history on a fishery. Incorporating life-history parameters into management and increasing legal size will improve sustainability of the golden king crab fishery and hopefully allow recovery of a collapsed fishery.

## Data accessibility

All data are available through the Dryad Digital Repository: doi:10.5061/dryad.jq344 [75].
